# Synthesis and biological evaluation of matrine derivatives containing benzo-α-pyrone structure as potent anti-lung cancer agents

**DOI:** 10.1038/srep35918

**Published:** 2016-10-27

**Authors:** Lichuan Wu, Guizhen Wang, Shuaibing Liu, Jinrui Wei, Sen Zhang, Ming Li, Guangbiao Zhou, Lisheng Wang

**Affiliations:** 1 School of Chemistry and Chemical Engineering, Guangxi University, Nanning, Guangxi 530004, PR China; 2 State Key Laboratory of Membrane Biology, Institute of Zoology, Chinese Academy of Sciences, Beijing, China; 3 Guangxi Scientific Research Center of Traditional Chinese Medicine, Guangxi University of Chinese Medicine, Nanning, Guangxi 530200, PR China; 4 Key Laboratory of Animal Models and Human Disease Mechanisms of the Chinese Academy of Sciences and Yunnan Province, Kunming Institute of Zoology, Kunming, Yunnan, People’s Republic of China

## Abstract

Matrine, an active component of root extracts from *Sophora flavescens Ait*, is the main chemical ingredient of Fufang Kushen injection which was approved by Chinese FDA (CFDA) in 1995 as an anticancer drug to treat non-small cell lung cancer and liver cancer in combination with other anticancer drugs. Owning to its druggable potential, matrine is considered as an ideal lead compound for modification. We delineate herein the synthesis and anticancer effects of 17 matrine derivatives bearing benzo-α-pyrone structures. The results of cell viability assays indicated that most of the target compounds showed improved anticancer effects. Further studies showed that compound **5i** could potently inhibit lung cancer cell proliferation *in vitro* and *in vivo* with no obvious side effects. Moreover, compound **5i** could induce G1 cell cycle arrest and autophagy in lung cancer cells through up-regulating P27, down-regulating CDK4 and cyclinD1 and attenuating *PI3K*/*Akt*/*mTOR* pathway. Suppression of autophagy attenuated **5i** induced proliferation inhibition. Collectively, our results infer that matrine derivative **5i** bears therapeutic potentials for lung cancer.

Lung cancer is the leading cause of cancer-related death, which has ranked first in men and second in women in morbidity and mortality^1^. Currently, chemotherapy is the main treatment of lung cancer^2^. Although the survival of lung cancer patients has been improved with the emergence of tyrosine kinase inhibitors^3^,^4^, there still comes with new issues, such as drug resistance. It is of significant need to develop novel drugs to improve lung cancer patient outcomes.

Matrine, extracted from roots of *Sophora Flavescens Ait* is the main chemical ingredient of Fufang Kushen injection which was approved by Chinese FDA (CFDA) in 1995 as an anticancer drug to treat non-small cell lung cancer and liver cancer in combination with other anticancer drugs^5^,^6^,^7^,^8^,^9^,^10^. Recently, considerable literatures have reported that matrine has promising anticancer activity^11^. Besides, due to its druggable advantages, such as flexibility structure and good safety profiles, matrine is considered as an ideal lead compound for further modification^12^,^13^,^14^,^15^.

In our previous study^16^, 19 matrine derivatives were synthesized and derivative **6i** showed the strongest anticancer effects towards A549 lung cancer cells *in vitro*. However, **6i** displayed obvious toxicity in the *in vivo* mouse model. In the present study, in order to obtain compounds with potent anticancer effects and low toxicity, we used matrine as a lead compound to synthesize 17 matrine derivatives bearing benzo-α-pyrone structures which are a group of compounds including flavonoids and coumarins and could improve the pharmacological activity of compounds^17^. Further, the anticancer effects of these 17 matrine derivatives were screened and the anticancer mechanism of compound **5i** was investigated.

## Results

### Cytotoxic activities of matrine derivatives

**Figure 1:**
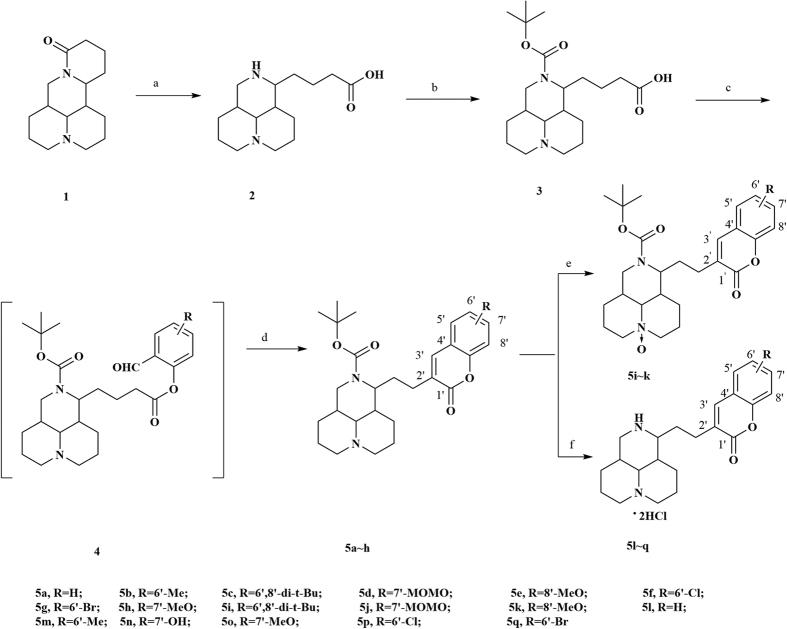
General synthetic route for target compounds. Reagents and conditions: (**a**) 20% aqueous KOH, reflux, 16 h; then 20% H_2_SO_4_; (**b**) (Boc)_2_O, Et_3_N, methanol, reflux, 3 h; (**c**) pivaloyl chloride, Et_3_N, DMAP, CH_2_Cl_2_, rt, overnight; (**d**) DBU, toluene, reflux, 32 h; (**e**) m-CPBA, CHCl_3_, 0 °C, 3 h; (f) HCl gas, PE/EtOAc (1:1 v-v), rt, 1 h.

The synthetic route for benzo-α-pyrone matrine derivatives is outlined in Fig. 1 and depicted in Methods and supplementary information. All the available derivatives were evaluated for their cytotoxic activities against four human cancer cell lines, including A549 (lung cancer cell), MCF-7 (breast cancer cell), SGC-7901 (gastric cancer cell) and Bel-7402 (hepatocellular cancer cell) (Table 1). Most of the derivatives exhibited improved anticancer efficacies with IC_50_ 15~484 times lower than that of matrine. Compound **5c**, **5f, 5g** and **5i** showed better anticancer effects than other matrine derivatives. The anticancer effectiveness of the original matrine compound and these four derivative compounds were ordered as follows: **5i** > **5c** > **5g** ≈ **5f** >> matrine. Compounds **5c** and **5i** have the same substituent group 6′,8′-di-tert-butyl in benzo-α-pyrone structure. Compound **5i** is the N-oxidation product of **5c**. It suggested that the substituent group 6′,8′-di-tert-butyl in benzo-α-pyrone structure and N-oxidation could both improve anticancer effects. It also can be found that 6′,8′-di-tert-butyl group showed better anticancer effects than 6′-bromo or 6′-chloro group in benzo-α-pyrone structure. Considering the favorable cytotoxic activities of compound **5i** and lung cancer morbidity and mortality, we chose compound **5i** as the representative compound for further investigation on lung cancer model.

### Compound 5i inhibits lung cancer cell proliferation and colony formation

**Figure 2:**
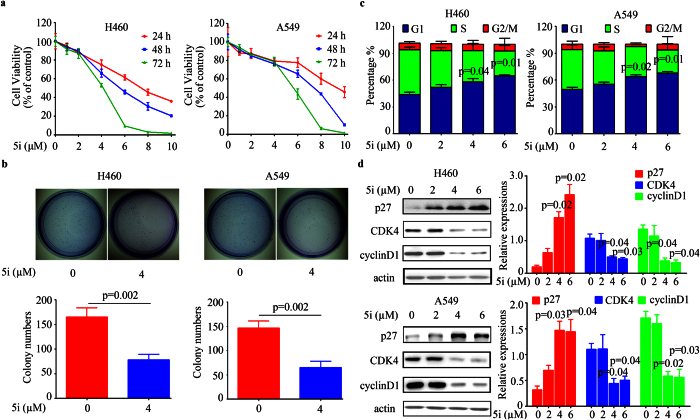
Compound 5i inhibits lung cancer cell proliferation, colony formation and induces G1 cell cycle arrest. (**a**) H460 and A549 cells were treated with different concentrations of **5i** for indicated time points and assessed by trypan blue exclusion analysis. (**b**) Soft-agar colony formation assay for H460 and A549 cells treated with or without **5i**. (**c**) **5i** induced G1 accumulation in a dose-dependent manner in H460 and A549 cells. (**d**) **5i** up-regulated P27 and down-regulated CDK4 and CyclinD1 in H460 and A549 cells. Data are represented as mean ± SD and p value was calculated with t-test.

To detect whether **5i** treatment could inhibit cell growth in lung cancer cells, cell viability assays were performed in lung cancer cells (Fig. S1). H1975, H460, and A549 were the most sensitive cell lines to compound **5i**. Furthermore, the results showed that **5i** potently inhibited cell growth in a time- and dose- dependent manner in the tested cell lines (Fig. 2a, Fig. S2a). The IC_50_ at 48 hours for A549, H460, and H1975 was found around 8, 6, and 5 μM, respectively. Therefore, we used 4 μM (around IC_20_) of **5i** in the following studies. The colony formation results demonstrated that **5i** significantly inhibited lung cancer cell colony forming activity (Fig. 2b, Fig. S2b).

### Compound 5i induces G1 cell cycle arrest in lung cancer cells

To further define the anticancer effects of **5i** on lung cancer cells, we conducted cell cycle analysis in A549, H460, and H1975 cells. Cells were treated with different concentrations of **5i** for 24 hours. The results displayed that **5i** arrested cell cycle of H460 and A549 at G1 phase in a dose-dependent manner (Fig. 2c). The percentage of G1 phase in H460 cells increased from 43% (control) to 57% (4 μM, p = 0.04) and 64% (6 μM, p = 0.01), respectively (Fig. 2c, left). Consistently, the percentage of G1 phase in A549 cells increased from 49% (control) to 63% (4 μM, p = 0.02) and 68% (6 μM, p = 0.01), respectively (Fig. 2c, right). However, **5i** could not induce G1 cell cycle arrest in H1975 cells (Fig. S2c). To dissect the underlying mechanism of compound **5i** induced G1 cell cycle arrest, we conducted western blot assays. The results revealed that upon **5i** treatment, CDK4 and CyclinD1 were down-regulated, while P27 was up-regulated in both H460 and A549 cells (Fig. 2d).

### Compound 5i induces autophagy and attenuates *PI3K*/*AKT*/*mTOR* pathway in lung cancer cells

**Figure 3:**
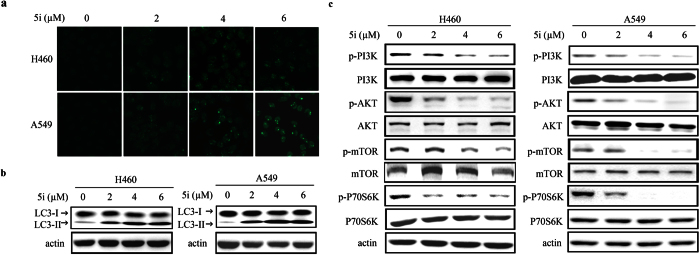
Compound 5i induces autophagy and attenuates *PI3K*/*Akt*/*mTOR* pathway in lung cancer cells. (**a**) H460 and A549 cells were treated with **5i** for 24 hours and assessed by immunofluorescence analysis using an anti-LC3 antibody. (**b**) H460 and A549 cells were treated with **5i** for 24 hours and the LC3 I and II levels were analyzed by western blotting. (**c**) **5i** attenuated *PI3K/Akt/mTOR* pathway in both H460 and A549 cells.

Autophagy is a lysosome-mediated process involved in protein and organelle degradation^18^. Autophagy could suppress tumor progression through limiting genome instability, restricting inflammation and promoting tumor cell apoptosis^19^. To test whether compound **5i** could induce autophagy, H460, A549, and H1975 cells were treated with **5i** for 24 hours. The immunofluorescence results exhibited that LC3, an autophagy indicator was activated in both H460 and A549 lung cancer cells (Fig. 3a). LC3 activation was also confirmed by western blotting assays results (Fig. 3b). While in H1975 cells, **5i** could not induce autophagy (Fig. S2d, S2e).

The process of autophagy is well-regulated and *PI3K*/*AKT*/*mTOR* pathway plays a key role in this process^18^,^20^. To further dissect the underlying molecular mechanism of compound **5i** induced autophagy, we detected the activity of *PI3K*/*AKT*/*mTOR* pathway in H460, A549, and H1975 lung cancer cells upon **5i** treatment. The results showed that with treatment of compound **5i**, the *PI3K*/*AKT*/*mTOR* pathway was down-regulated in both H460 and A549 lung cancer cells in a dose-dependent manner (Fig. 3c), but not in H1975 lung cancer cells (Fig. S2e).

### Suppression of autophagy attenuates compound 5i induced cell viability inhibition and G1 cell cycle arrest

**Figure 4:**
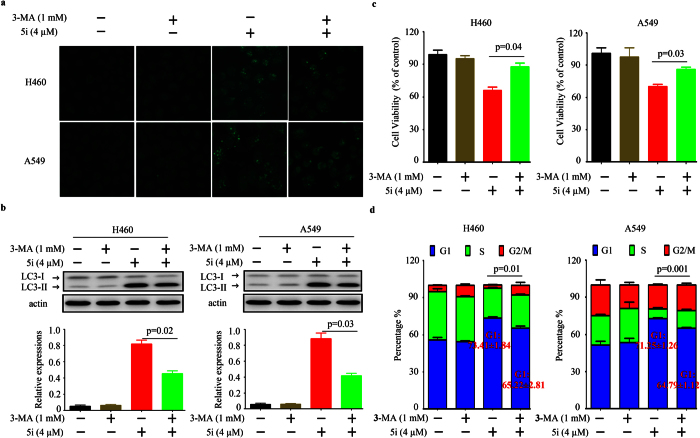
Suppression of autophagy by 3-MA attenuates 5i induced cell viability inhibition. (**a**) Co-treatment of 3-MA with **5i** in H460 and A549 cells and immunofluorescence assays for detecting LC3 I and LC3 II. (**b**) Co-treatment of 3-MA with **5i** in H460 and A549 cells and western blotting assays for detecting LC3 I and LC3 II expression. (**c**) Co-treatment of 3-MA with **5i** in H460 and A549 cells and MTT assays for detecting viable cells. (**d**) Co-treatment of 3-MA with **5i** in H460 and A549 cells and G1 cell cycle distribution. Data are represented as mean ± SD and p value was calculated with t-test.

To unveil the role of autophagy in **5i** induced cell viability inhibition, we alleviated autophagy induced by **5i** by using autophagy inhibitor 3-MA and evaluated the cell viabilities in H460 and A549 cells. The results showed that with co-treatment of **5i** and 3-MA, **5i** induced autophagy was attenuated by 3-MA reflected by immunofluorescence (Fig. 4a) and western blotting results (Fig. 4b). Moreover, the results of MTT assays showed that the cell viability inhibition of **5i** was also attenuated by 3-MA in both H460 and A549 lung cancer cells (Fig. 4c). Further investigations on the G1 cell cycle indicated that co-treatment of **5i** with 3-MA significantly alleviated the G1 cell cycle arrest in both H460 and A549 lung cancer cells (Fig. 4d).

### *In vivo* anti-lung cancer activity of compound 5i

**Figure 5:**
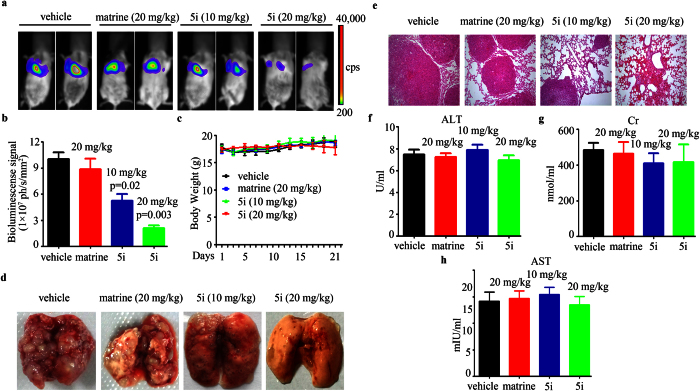
*In vivo* anti-lung cancer effects of 5i. (**a**) The mice were detected by IVIS Spectrum. (**b**) The relative luciferase intensity in the mice. (**c**) The body weight of mice was monitored every two days. (**d**) Representative images of dissected lung tissue from each group. (**e**) Hematoxylin and eosin (HE) staining of lung tissue sections of mice from each group. (**f–h**) The serum ALT (**f**), Cr (**g**), and AST (**h**) levels were detected of mice from each group. Data are represented as mean ± SD and p value was calculated with t-test.

To evaluate the anti-lung cancer activity of compound **5i** *in vivo*, A549-luciferase cells (1 × 10^6^) were intravenously injected into SCID/Beige mice (n = 6 for each group). Vehicle, **5i** (10, 20 mg/kg), and matrine (20 mg/kg) were intraperitoneally administrated every other days for 3 weeks. The results demonstrated that **5i** significantly suppressed tumor growth reflected by decrease of luciferase bioluminescence intensity, while matrine had no obvious effect (Fig. 5a,b). Besides, **5i** treatments did not lead to body weight reduction (Fig. 5c). Tumor can be obviously found in the dissected lung tissue of vehicle and matrine group, while in **5i** group, tumor size decreased dramatically in a dose-dependent manner (Fig. 5d). Consistent with the results in Fig. 5a,d, 5**i** reduced dissemination of disease and prevented destruction of tissue architectures reflected by HE staining (Fig. 5e). We also tested the adverse effects of **5i**. The results demonstrated that mice treated with **5i** had normal serum concentration of Alanine Aminotransferase (ALT), creatinine (Cr), and Aspartate aminotransferase (AST) compared with vehicle control (Fig. 5f–h). These results inferred that **5i** displayed favorable anti-tumor effect *in vivo* with no obvious side effects.

## Discussion

Matrine is a highly polar basic compound and used in the treatment of hepatitis and hepatic fibrosis in China for a long time with low toxicity^21^. Recently, the anticancer effects of matrine have become attractive for its broad anticancer spectrum and good safety^22^,^23^,^24^. Besides, clinical studies have demonstrated that the quality of life and immune function of cancer patients were largely improved by combining standard therapies with the use of matrine^25^,^26^. However, the low bioavailability of matrine has limited its use as an anticancer drug. In virtue of its favorable safety and low bioactivity, we designed and synthesized 17 matrine derivatives bearing benzo-α-pyrone structure which appears as a core skeleton in many anticancer compounds^17^. The results of *in vitro* cytotoxic activity assays indicated that most of the target compounds showed improved anticancer effects with IC_50_ 15~484 times lower than that of matrine in four tested human cancer cell lines (Table 1). Compound **5i** exhibited the most potent anticancer effects. Moreover, compound **5i** inhibited lung cancer cell proliferation *in vitro* and *in vivo* (Fig. 2a, Fig. S2a, and Fig. 5a,b). Interestingly, in our previous study, we discovered that matrine derivative **6i** bearing *p*-methoxyphenyl structure also showed strong anticancer effect in A549 lung cancer cell lines (IC_50_ = 1.6 μM), but displayed toxicity in the *in vivo* mouse model. Compared with **6i**, compound **5i** of this study displayed no obvious side effects reflected by body weight loss and ALT, AST and Cr detection (Fig. 5c,f–h). These results implied that compound **5i** displayed advantage in drug safety and druggable potential.

Epidermal growth factor receptor (EGFR) mutation plays an oncogenic role in lung cancer initiation^27^. Lung cancer patients with EGFR mutation accounts for 10% of non-small cell lung cancer (NSCLC) in United States and about 40% of NSCLC in East Asia^4^,^28^. Thus, tyrosine kinase inhibitors (TKIs) specific for EGFR (EGFR-TKIs) have become a main focus in lung cancer therapy. The efficiency of first-generation EGFR-TKIs, such as gefitinib, could reach 70–80% in NSCLC patients harboring EGFR mutations (exon 19 deletion and L858R)^4^,^29^. However, patients gradually develop acquired resistance to EGFR-TKIs within 12 months. The most common mechanism of resistance is a second mutation of EGFR (T790M), which accounts for 50% of all resistances cases and results in the continued activation of *PI3K*/*AKT* pathway^30^. Thus, the therapeutic strategies for lung cancer patients with EGFR wild type, first mutation, or acquired mutation could be different. In this study, we discovered that compound **5i** could not only inhibit the proliferation of A549 and H460 lung cancer cells (EGFR wild type) but also H1975 lung cancer cells (EGFR acquired mutation, L858R and T790M mutation) (Fig. 2a, Fig. S2a). However, compound **5i** could induce G1 cell cycle arrest and autophagy and attenuate *PI3K*/*AKT* pathway in A549 and H460 lung cancer cells (Fig. 2c, Fig. 3) but not in H1975 lung cancer cells (Fig. S2c–e). The anti-proliferative activity mechanism of **5i** on EGFR wild type lung cancer cells and EGFR double mutation lung cancer cells might be different. Thus, the anti-proliferative effects mechanism of **5i** on H1975 lung cancer cells needs further investigated.

Autophagy is a cellular process whereby the cell degrades subcellular materials to generate energy. To our best knowledge, autophagy plays a paradoxical role in cancer development^18^. Inactivation of autophagy-specific genes (*beclin1*, *atg5*) resulted in increased tumorigenesis while activation of autophagy may help cancer cells survive in nutrient-limited environments. Therefore, it is important to distinguish between cytoprotective and cytotoxic autophagy. In this study, we found that compound **5i** could induce autophagy in lung cancer cells (Fig. 3). Moreover, suppression of autophagy with 3-MA attenuated **5i** induced cell viability inhibition (Fig. 4c). These results indicated that autophagy played a cytotoxic role in compound **5i** induced lung cancer cell viability inhibition. *PI3K*/*Akt* pathway is abnormally activated in many malignancies (such as gastric, breast and hepatic cancer), which could turn autophagy off^20^. Also, *PI3K*/*Akt* pathway plays a vital role in cancer cell proliferation. In this study, we discovered that compound **5i** could dramatically down-regulated *PI3K*/*Akt* pathway. Considering abnormal activities of *PI3K*/*Akt* pathway in cancer and the inhibition effects of **5i** on *PI3K*/*Akt* pathway, we could infer that **5i** exhibited its pan anticancer effects by inhibiting *PI3K*/*Akt* pathway activities.

In conclusion, we synthesized 17 matrine derivatives which showed improved anticancer activities towards cancer cell lines. Compound **5i** displayed the strongest anticancer activity which inhibited lung cancer cell proliferation *in vitro* and *in vivo* with no obvious side effects. Further studies indicated that compound **5i** arrested cell cycle at G1 phase and induced autophagy in lung cancer cells. Moreover, compound **5i** could down-regulate *PI3K*/*Akt* pathway and suppression of autophagy attenuated **5i** induced proliferation inhibition. Our studies suggested that matrine derivative **5i** could be a potential effective compound to treat lung cancer.

## Methods

### General procedure of matrine derivatives synthesis

Firstly, matrine was hydrolyzed with aqueous potassium hydroxide to produce matrine acid (intermediate **2**). Then in the presence of triethylamine (Et_3_N), intermediate **3** was prepared via the reaction of intermediate **2** with di-tert-butyl dicarbonate ((Boc)_2_O) in the reflux of methanol^31^. Intermediate **4** was obtained through the reaction of intermediate **3** with corresponding salicylaldehydes by mixed anhydrides method using 4-dimethylamiopryidine (DMAP) as catalyst^32^. In this step, 4-hydroxy salicylaldehyde was selectively protected by methoxymethyl ether^33^. Products **5a~h** were prepared via intramolecular aldol reaction with 1,8-diazabicyclo[5.4.0]undec-7-ene (DBU) in refluxing anhydrous toluene^34^. In view of natural prevalently occurring N-oxidation in quinolizidine alkaloid, derivatives **5i~k** were synthesized by converting products **5c~e** to their N-oxide forms with 3-chloroperbenzoic acid (m-CPBA) in ice-cold chloroform. Derivatives **5l~q** were produced by removing the N-Boc structure from their corresponding products **5** in hydrochloric acid methanol solution rather than trifluoroacetic acid/dichloromethane concerning to the lactone stability. Under this acidic condition, the protection group methoxymethyl ether of **5d** was dropped off along with N-Boc, giving the corresponding phenolic compound.

All reagents and solvents were purchased from commercial sources. Further purification and drying by standard methods were employed when necessary. For thin layer chromatography (TLC) analysis Qingdao haiyang GF254 silica gel plates were used. Column chromatography was carried out using Qingdao haiyang 300–400 mesh silica gel. All NMR spectra were recorded on Bruker AVANCE 600 spectrometers operating at ^1^H and ^13^C frequencies of 600.17 and 150.91 MHz, respectively, using DMSO-*d*_6_ or CDCl_3_ as solvent. Chemical shifts (δ) are in ppm relative to the residual solvent signal (DMSO-*d*_6_ with 2.48 and 39.52 ppm and CDCl_3_ with 7.26 and 77.16 ppm for ^1^H and ^13^C, respectively). The coupling constant (J) was presented in hertz (Hz). Electrospray ionization mass spectra (ESI-MS) were recorded on a Thermo Scientific TSQ Quantum Access Max mass spectrometer.

### Cell culture, cell proliferation and cell viability assays

Human cancer cell lines used in this study were purchased from ATCC (American Type Culture Collection) and cultured in DMEM high-glucose (Invitrogen, Carlsbad, CA, USA) supplemented with 10% fetal bovine serum (FBS) and 1% penicillin-streptomycin. Cells were cultured at 37 °C, in an atmosphere of 95% air and 5% CO_2_ under humidified condition^35^,^36^. For MTT assay, cells (5000 cells per well) were plated in flat-bottomed 96-well micro plates. Sixteen hours after seeding, new medium containing different concentrations of target compounds or solvent control (DMSO) was added. Cells were further incubated for indicated times and incubated with MTT for additional 2–4 hours. The plates were then assayed by testing the absorbance at 490 nm. Cell viability was estimated by trypan blue dye exclusion assay^37^.

### Clonogenic assay and cell cycle analysis

For clonogenic assay, the cells were suspended in 1 ml DMEM containing 0.3% low-melting-point agarose (Amresco, Solon, OH) and 10% FBS, and plated on a bottom layer containing 0.6% agarose and 4 μM **5i** in 35 mm plates (1000 cells/plate). After 14 days of culture, cells were stained with Giemsa and clones containing more than 50 cells were counted. For cell cycle analysis, the cells were treated with **5i** at different concentrations for 24 hours. Then cells were digested with 0.25% trypsin and washed with cold PBS for 2–3 times. Later, cells were fixed with 70% ethanol at −20 °C overnight. DNA content was determined by PI staining and flow cytometry analysis.

### Immunofluorescence analysis

Cells (2 × 10^5^ per well) were seeded on cover-slips with 1% gelatin in 6-well culture plates and treated with **5i** (2, 4 and 6 μM) for 24 hours. The cells were then washed with cold PBS and fixed with 4% paraformaldehyde at room temperature for 15 min. The cells were washed and blocked by incubating with 5% BSA (Ameresco, USA) in PBS and incubated with an anti-LC3 antibody overnight at 4 °C, followed by incubation with a FITC-conjugated secondary antibody for 2 hours at room temperature in a dark humid chamber. Following washed with Tween-20/PBS for 3 times, the cover-slips were inverted on the glass slides. The cells were observed under the confocal microscope (Zeisis LSM 510 Meta).

### ELISA

Mouse blood was obtained before cervical dislocation. Serum was separated by centrifugation at 2000 g and concentration of ALT, Cr, and AST in serum of mice was determined by ELISA using commercially available ELISA kit (TSZ) according to the manufacture instructions. The absorbance of the plates was read at 450 nm using an automated microplate reader (Bio-Tek, Winooski, VT, USA).

### Western blotting

Cells were lysed in RIPA buffer supplemented with protease inhibitors. Proteins (20 μg) were subjected to 6–15% SDS-PAGE, electrophoresed and transferred on to a nitrocellulose membrane. After blocking with 5% non-fat milk in Tris-buffered saline, the membrane was washed and incubated with the indicated primary and secondary antibodies and detected using the Luminescent Image Analyser LSA 4000 (GE, Fairfield, CO, USA).

### Murine models

The animal studies were approved by the Institutional Review Board of Institute of Zoology, Chinese Academy of Sciences. All animal studies were conducted according to protocols approved by the Animal Ethics Committee of the Institute of Zoology, Chinese Academy of Sciences. SCID/beige mice were injected with A549-luciferase (A549-Luc) cells (1 × 10^6^) via tail vein, and 4 days later the mice were randomized into 4 groups to receive treatment with intraperitoneal injection of vehicle, matrine at 20 mg/kg, **5i** at 10 and 20 mg/kg (*n* = 6 for each group; once every two days for 21 days). The mice were imaged by IVIS Spectrum at day 21, and were euthanized by cervical dislocation.

### Statistical analysis

The data are presented as the mean ± SD of three independent experiments. Differences between data groups were evaluated for significance using Student’s t-test of unpaired data (two-tailed). P values less than 0.05 were considered statistically significant in all cases.

## Additional Information

**How to cite this article**: Wu, L. *et al*. Synthesis and biological evaluation of matrine derivatives containing benzo-α-pyrone structure as potent anti-lung cancer agents. *Sci. Rep.* **6**, 35918; doi: 10.1038/srep35918 (2016).

**Publisher’s note:** Springer Nature remains neutral with regard to jurisdictional claims in published maps and institutional affiliations.

## Supplementary Material

Supplementary Information

## Figures and Tables

**Table 1:** Anti-proliferative activities of target compounds in human cancer cell lines.

	IC_50_ (μM)			
Compounds	SGC-7901	MCF-7	A549	Bel-7402
5a	58.5 ± 4.2	81.7 ± 1.2	83.2 ± 5.6	61.4 ± 7.7
5b	47.3 ± 4.5	35.2 ± 3.8	68.9 ± 11.5	55.8 ± 4.1
5c	13.2 ± 2.8	14.2 ± 3.2	18.0 ± 4.2	15.6 ± 8.4
5d	71.8 ± 5.8	82.9 ± 1.9	>100	69.4 ± 5.1
5e	52.8 ± 2.0	59.9 ± 4.0	70.3 ± 9.1	63.1 ± 6.3
5f	30.3 ± 2.6	33.8 ± 2.5	38.4 ± 2.8	27.8 ± 3.1
5g	26.7 ± 2.1	29.2 ± 1.8	34.7 ± 4.2	32.2 ± 3.5
5h	55.2 ± 3.7	53.4 ± 4.5	65.1 ± 7.1	71.1 ± 6.7
5i	9.4 ± 1.9	7.3 ± 0.5	8.1 ± 0.3	7.6 ± 0.8
5j	63.3 ± 4.6	55.5 ± 5.4	89.9 ± 9.6	63.7 ± 4.9
5k	40.9 ± 3.7	55.1 ± 5.3	60.2 ± 5.4	63.8 ± 4.9
5l	88.0 ± 8.2	90.1 ± 6.2	>100	87.8 ± 9.7
5m	62.4 ± 4.2	45.2 ± 4.4	86.8 ± 7.6	64.7 ± 7.5
5n	94.9 ± 4.8	99.1 ± 5.6	>100	88.2 ± 5.9
5o	87.7 ± 7.8	62.2 ± 3.9	94.7 ± 9.2	74.3 ± 8.5
5p	76.2 ± 6.0	56.1 ± 3.6	93.5 ± 10.1	63.1 ± 5.9
5q	39.9 ± 1.9	33.3 ± 1.9	52.9 ± 2.5	40.8 ± 2.2
matrine	1380 ± 116	2497 ± 208	3923 ± 243	2057 ± 192

Note: IC_50_ values are taken as a mean from 3 experiments. Mean ± SD.
