# Supplementary material for: Synthesis and biological evaluation of matrine derivatives containing benzo-α-pyrone structure as potent anti-lung cancer agents
Source: Sci Rep. 2016 Oct 27;6:35918. doi: 10.1038/srep35918 (PMC5081519; doi:10.1038/srep35918)
Supplement: Supplementary Information [file srep35918-s1.pdf]

Synthesis and biological evaluation of matrine derivatives containing benzo-α-pyrone structure as potent anti-lung cancer agents

Lichuan Wu[a,1], Guizhen Wang[b,1], Shuaibing Liu[a,1], Jinrui Wei[c,1], Sen Zhang[a], Ming Li[d], Guangbiao Zhou[b,*], Lisheng Wang[a,*]

Supplementary Information

Synthesis of intermediate 2

Matrine (10 mmol) was added to a stirred 10% aqueous KOH solution (40 mL) and the mixture was stirred at reflux for 16 hours. The reaction mixture was cooled to room temperature and then was acidified in ice bath with 20% aqueous H_2SO_4 till pH value reached to 7~8. The solvent was removed in vacuo, and the resulting solid was dissolved in methanol (MeOH) followed by filtration. The filtrate was concentrated under reduced pressure to a small volume. Then the product was obtained by precipitation with acetone followed by filtration as a white solid in 89% yield.

Synthesis of intermediate 3

Et_3N (15.0 mmol) was added into a stirred solution of the intermediate 2 (5.0 mmol) dissolved in methanol (30 mL) and the mixture was heated to reflux. $(Boc)_2O$ (10 mmol) was then added dropwise and the reaction mixture was stirred at reflux for 3 hours. The solvent was evaporated under reduced pressure, and then was dissolved in 1 M NaOH (30 mL). The resulting aqueous phase was washed with diethyl ether and then was acidified with 6 N HCl till pH value reached to 6. Then the solvent was removed in vacuo and the solid residue was dissolved in CH_2Cl_2 (100 ml) followed by

filtration. The filtrate was concentrated under reduced pressure to obtain the desired product as light brown oil in 80% yield.

Synthesis of intermediates 4a~4h

The intermediate **3** (10 mmol) was firstly dissolved in dichloromethane (50 mL) followed an adding of freshly distilled Et_3N (3 eq.) and the mixture was cooled to 0 ℃ in ice bath. Pivaloyl chloride was added dropwise followed by keeping the temperature for 20 minutes. The appropriate salicylaldehyde (1.5 eq.) and DMAP (0.01 eq.) were added and the reaction mixture was stirred overnight at room temperature. The solution was washed with water and brine, and the organic phase was concentrated under reduced pressure. The residue was suspended in 1 N HCl (40 mL) and the turbid solution was washed with ethyl ether as quickly as possible. The clarified water phase was alkalified with saturated aqueous Na_2CO_3 till the pH value reached to 8. The resulting emulsion was then extracted with ethyl ether. The combined organic layers were dried over anhydrous Na_2SO_4, filtered, and the solvent was removed to obtain the crude 2-formyl phenyl ester. These crude ester were identified with both positive Dragendorff's reagent reaction and fluorescence quenching at 254 nm by TLC analysis using developing solvent PE/ EtOAc (1:1 v: v), and were used in the next step without further purification.

Synthesis of derivatives 5a~5h

A crude of 2-formyl phenyl ester 4 (2.8 g) was dissolved in dry toluene (30 mL). Then DBU was added in equivalent weight and the mixture was stirred at reflux for 32 hours. The reaction mixture was diluted with ethyl acetate (40 mL), and washed with

water till aqueous layer was colorless. The organic layers were dried over anhydrous $NaSO_4$, filtered, and the solvent was removed in vacuo. The residue was purified by column chromatography eluting with PE/EtOAc (9:1 to 4:1 v: v) to obtain the pure products.

tert-butyl 1-(2-(2-oxo-2H-chromen-3-yl)ethyl)octahydro-1H,4H-pyrido[3,2,1-ij][1,6]naphthyridine-2(3H)-carboxylate **(5a, $C_{27}H_{36}N_2O_4$)**

Light brown viscous oil; two-step yield: 30%; ^1H NMR ($CDCl_3$, 600 MHz): δ 7.51 (s, 1H), 7.39-7.46 (m, 2H), 7.29 (d, J = 8.2 Hz, 1H), 7.21-7.24 (m, 1H), 3.81-3.87 (m, 1H), 3.51 (dd, J = 8.8 Hz, 13.6 Hz, 1H), 3.38 (dd, J = 7.2 Hz, 13.6 Hz, 1H), 2.62-2.70 (m, 3H), 2.52-2.58 (m, 1H), 2.04-2.14 (m, 1H), 1.97 (t, J = 3.1 Hz, 1H), 1.62-1.86 (m ,8H), 1.51-1.58 (m, 2H), 1.28-1.46 (m, 13H); ^{13}C NMR ($CDCl_3$, 151 MHz): δ 161.92, 156.03, 153.34, 139.00, 130.51, 129.98, 127.30, 124.28, 119.84, 116.48, 79.21, 63.52, 57.04, 56.92, 54.89, 44.97, 40.56, 35.27, 30.48, 29.10, 28.67(3C), 28.66, 28.28, 21.35, 21.22. ESI-MS: $[M+1]^+$, 452.89.

tert-butyl 1-(2-(6-methyl-2-oxo-2H-chromen-3-yl)ethyl)octahydro-1H,4H-pyrido[3,2,1-ij][1,6]naphthyridine-2(3H)-carboxylate **(5b, $C_{28}H_{38}N_2O_4$)**

Light brown viscous oil; two-step yield: 30%; ^1H NMR ($CDCl_3$, 600 MHz) : δ 7.46 (s, 1H), 7.23-7.26 (m, 1H), 7.18-7.21 (m, 2H), 3.80-3.86 (m, 1H), 3.49 (dd, J = 8.9 Hz, 13.6 Hz, 1H), 3.39 (dd, J = 7.0 Hz, 13.6 Hz, 1H), 2.61-2.71 (m, 3H), 2.50-2.57 (m, 1H), 2.38 (s, 3H), 2.03-2.13 (m, 1H), 1.97 (t, J = 3.2 Hz, 1H), 1.61-1.86 (m ,8H), 1.51-1.59 (m, 2H), 1.29-1.49 (m, 13H); ^{13}C NMR ($CDCl_3$, 151 MHz): δ ppm 162.06, 155.94, 151.34, 138.94, 133.78, 131.39, 129.67, 127.04, 119.46, 116.08, 79.10, 63.43,

56.93, 56.81, 54.79, 44.92, 40.42, 35.18, 30.34, 28.97, 28.54, 28.13, 21.23, 21.12, 20.78. ESI-MS: $[M+1]^+$, 466.81.

tert-butyl 1-(2-(6,8-di-tert-butyl-2-oxo-2H-chromen-3-yl)ethyl)octahydro-1H,4H-pyrido[3,2,1-ij][1,6]naphthyridine-2(3H)-carboxylate **(5c, $C_{35}H_{52}N_2O_4$)**

Light brown viscous oil; two-step yield: 30%; ^{1}H NMR ($CDCl_3$, 600 MHz): δ 7.49 (s, 1H), 7.48 (d, J = 2.3 Hz, 1H), 7.24 (d, J = 2.3 Hz, 1H), 3.82-3.89 (m, 1H), 3.52 (dd, J = 8.9 Hz, 13.6 Hz, 1H), 3.42 (dd, J = 7.1 Hz, 13.6 Hz, 1H), 2.61-2.72 (m, 3H), 2.50-2.57 (m, 1H), 2.06-2.16 (m, 1H), 1.98 (t, J = 3.1 Hz, 1H), 1.63-1.86 (m ,8H), 1.53-1.61 (m, 2H), 1.48-1.53 (s, 9H), 1.28-1.49 (m, 22H); ^{13}C NMR ($CDCl_3$, 151 MHz): δ ppm 161.73, 155.93, 149.88, 146.30, 140.19, 136.84, 128.58, 125.57, 121.90, 119.51, 79.07, 63.47, 56.96, 56.84, 54.94, 40.48, 35.15, 35.06, 34.63, 31.43, 31.32, 30.56, 29.95, 29.28, 28.98, 28.57, 28.39, 28.19, 21.27, 21.13. ESI-MS: $[M+1]^+$, 564.90.

tert-butyl 1-(2-(7-(methoxymethoxy)-2-oxo-2H-chromen-3-yl)ethyl)octahydro-1H,4H-pyrido[3,2,1-ij][1,6]naphthyridine-2(3H)-carboxylate **(5d, $C_{29}H_{40}N_2O_6$)**

Light brown viscous oil; two-step yield: 30%; ^{1}H NMR ($CDCl_3$, 600 MHz): δ ppm 7.45 (s, 1H), 7.32 (d, J = 8.5 Hz, 1H), 6.98 (d, J = 2.3 Hz, 1H), 6.92 (dd, J = 2.3 Hz, 8.5 Hz, 1H), 5.21 (s, 2H), 3.79-3.85 (m, 1H), 3,45-3.51 (m, 4H), 3.38 (dd, J = 7.1 Hz, 13.6 Hz, 1H), 2.59-2.70 (m, 3H), 2.46-2.54 (m, 1H), 2.01-2.11 (m, 1H), 1.97 (t, J = 3.2 Hz, 1H), 1.61-1.87 (m ,8H), 1.51-1.58 (m, 2H), 1.22-1.49 (m, 13H); ^{13}C NMR ($CDCl_3$, 151 MHz) δ 194.61, 162.05, 159.17, 155.93, 154.52, 138.94, 135.38, 128.04, 126.77, 114.27, 113.28, 109.07, 103.48, 103.40, 94.44, 94.07, 79.12, 63.41, 58.45,

56.92, 56.80, 56.28, 54.74, 44.85, 40.41, 35.13, 30.41, 28.96, 28.56, 28.37, 28.15, 21.22, 21.10, 18.43. ESI-MS: $[M+1]^+$, 512.81.

tert-butyl 1-(2-(8-methoxy-2-oxo-2H-chromen-3-yl)ethyl)octahydro-1H,4H-pyrido[3,2,1-ij][1,6]naphthyridine-2(3H)-carboxylate **(5e, $C_{28}H_{38}N_2O_5$)**

Light brown viscous oil; two-step yield: 30%; ^{1}H NMR ($CDCl_3$, 600 MHz): δ 7.49 (s, 1H), 7.16 (t, J = 7.9 Hz, 1H), 6.98-7.02 (m, 2H), 3.78-3.84 (m, 1H), 3.46 (dd, J = 8.9 Hz, 13.6 Hz, 1H), 3.38 (dd, J = 7.1 Hz, 13.6 Hz, 1H), 2.62-2.70 (m, 3H), 2.51-2.58 (m, 1H), 2.02-2.12 (m, 1H), 1.96 (t, J = 3.1 Hz, 1H), 1.60-1.87 (m ,8H), 1.51-1.58 (m, 2H), 1.28-1.48 (m, 13H); ^{13}C NMR ($CDCl_3$, 151 MHz): δ ppm 161.33, 156.05, 147.08, 142.94, 139.19, 130.15, 124.13, 120.43, 118.86, 112.49, 79.22, 63.52, 57.02, 56.90, 56.30, 54.86, 45.09, 40.50, 35.28, 30.24, 29.04, 28.66 (3C), 28.60, 28.18, 21.30, 21.21. ESI-MS: $[M+1]^+$, 482.81.

tert-butyl 1-(2-(6-chloro-2-oxo-2H-chromen-3-yl)ethyl)octahydro-1H,4H-pyrido[3,2,1-ij][1,6]naphthyridine-2(3H)-carboxylate **(5f, $C_{27}H_{35}ClN_2O_4$)**

Light brown viscous oil; two-step yield: 30%; ^{1}H NMR ($CDCl_3$, 600 MHz): δ 7.44 (s, 1H), 7.37-7.41 (m, 2H), 7.24 (d, J = 8.46 Hz, 2H), 3.78-3.85 (m, 1H), 3.48 (dd, J = 8.9 Hz, 13.6 Hz, 1H), 3.40 (dd, J = 7.0 Hz, 13.6 Hz, 1H), 2.63-2.71 (m, 3H), 2.51-2.58 (m, 1H), 2.06-2.16 (m, 1H), 1.98 (t, J = 3.2 Hz, 1H), 1.60-1.87 (m ,8H), 1.51-1.60 (m, 2H), 1.28-1.47 (m, 13H); ^{13}C NMR ($CDCl_3$, 151 MHz) δ 161.25, 155.92, 151.54, 137.71, 131.16, 130.33, 129.39, 126.46, 120.75, 117.77, 79.25, 63.45, 58.37, 56.90, 56.79, 54.82, 45.11, 40.35, 35.21, 30.09, 28.85, 28.53, 28.03, 21.17, 21.07, 18.38. ESI-MS: $[M+1]^+$, 486.76**.**

tert-butyl 1-(2-(6-bromo-2-oxo-2H-chromen-3-yl)ethyl)octahydro-1H,4H-pyrido [3,2,1-ij][1,6]naphthyridine-2(3H)-carboxylate **(5g, $C_{27}H_{35}BrN_2O_4$)**

Light brown viscous oil; two-step yield: 30%; ^{1}H NMR ($CDCl_3$, 600 MHz): δ ppm 7.55 (d, J = 2.3 Hz, 1H), 7.53 (dd, J = 2.3 Hz, 8.7 Hz, 1H), 7.44 (s, 1H), 7.18 (d, J = 8.7 Hz, 1H), 3.78-3.85 (m, 1H), 3.48 (dd, J = 9.0 Hz, 13.5 Hz, 1H), 3.40 (dd, J = 6.9 Hz, 13.5 Hz, 1H), 2.63-2.72 (m, 3H), 2.51-2.58 (m, 1H), 2.05-2.15 (m, 1H), 1.98 (t, J = 3.0 Hz, 1H), 1.60-1.88 (m ,8H), 1.52-1.60 (m, 2H), 1.28-1.50 (m, 13H); ^{13}C NMR ($CDCl_3$, 151 MHz): δ ppm 161.15, 155.92, 152.05, 137.56, 133.16, 131.23, 129.51, 121.28, 118.12, 116.73, 79.19, 63.46, 56.93, 56.82, 54.84, 40.39, 35.26, 30.09, 28.91, 28.61, 28.56, 28.06, 21.22, 21.13. ESI-MS: $[M+1]^+$, 532.70.

tert-butyl 1-(2-(7-methoxy-2-oxo-2H-chromen-3-yl)ethyl)octahydro-1H,4H-pyrido[3,2,1-ij][1,6]naphthyridine-2(3H)-carboxylate **(5h, $C_{28}H_{38}N_2O_5$)**

Light brown viscous oil; two-step yield: 30%; ^{1}H NMR ($CDCl_3$, 600 MHz): δ 7.46 (s, 1H), 7.31 (d, J = 8.4 Hz, 1H), 6.79-6.83 (m, 2H), 3.85 (s, 3H), 3.79-3.84 (m, 1H), 3.49 (dd, J = 8.9 Hz, 13.6 Hz, 1H), 3.39 (dd, J = 7.1 Hz, 13.6 Hz, 1H), 2.59-2.70 (m, 3H), 2.47-2.54 (m, 1H), 2.02-2.12 (m, 1H), 1.96 (t, J = 3.2 Hz, 1H), 1.61-1.87 (m ,8H), 1.51-1.59 (m, 2H), 1.29-1.49 (m, 13H); ^{13}C NMR ($CDCl_3$, 151 MHz) δ 162.18, 161.74, 139.11, 128.05, 126.13, 113.33, 112.28, 100.45, 79.11, 63.41, 56.91, 56.79, 55.70, 54.75, 40.40, 35.12, 30.45, 28.95, 28.56, 28.33, 28.14, 21.21, 21.09. ESI-MS: $[M+1]^+$, 482.80.

Synthesis of derivatives 5i~5k

Products **5** (0.85 mmol) were dissolved in $CHCl_3$ (20 mL). Then m-CPBA (1.5 eq.)

was added and the mixture was stirred at 0 ℃ for 3 hours. The solvent was removed under reduced pressure. The residue was purified by column chromatography eluting with MeOH/AcOEt (1:4 to 1:2 v: v) to obtain pure products.

2-(tert-butoxycarbonyl)-1-(2-(6,8-di-tert-butyl-2-oxo-2H-chromen-3-yl)ethyl)decahydro-1H-pyrido[3,2,1-ij][1,6]naphthyridine 7(3a1H)-oxide **(5i, $C_{35}H_{52}N_2O_5$)**

White foam; 91% yield form 5c; ^{1}H NMR ($CDCl_3$, 600 MHz): δ ppm 7.50 (s, 1H), 7.47 (d, J = 2.2 Hz, 1H), 7.24 (d, J = 2.2 Hz, 1H), 5.03 (br s, 1H), 4.14-4.25 (m,1H), 3.59-3.66 (m, 1H), 3.44 (br s, 2H), 3.06-3.20 (m, 3H), 2.50-2.74 (m, 4H), 2.14-2.24 (m, 1H), 1.74-1.81 (m, 1H), 1.65-1.74 (m, 1H), 1.62-1.76 (m, 2H), 1.41-1.62 (m,21H), 1.33 (s, 9H); ^{13}C NMR ($CDCl_3$, 151 MHz): δ ppm 161.80, 156.65, 149.84, 146.31, 140.13, 136.94, 136.80, 129.73, 128.72, 127.59, 125.60, 121.93, 119.51, 79.68, 54.87, 35.05, 34.63, 31.44, 30.27, 29.96, 28.56, 27.72, 25.80, 25.21, 17.30, 17.23. ESI-MS: $[M+1]^+$, 580.79.

2-(tert-butoxycarbonyl)-1-(2-(7-(methoxymethoxy)-2-oxo-2H-chromen-3-yl)ethyl)decahydro-1H-pyrido[3,2,1-ij][1,6]naphthyridine 7(3a1H)-oxide **(5j, $C_{29}H_{40}N_2O_7$)**

White foam; 89% yield form 5d; ^{1}H NMR ($CDCl_3$, 600 MHz): δ ppm 7.46 (s, 1H), 7.34 (d, J = 8.6 Hz, 1H), 6.96 (d, J = 2.3 Hz 1H), 6.91 (dd, J = 2.3 Hz, 8.6 Hz 1H), 5.02 (br s, 1H), 4.14 (t, J = 12.2 Hz, 1H), 3.60 (dd, J = 5.2 Hz, 12.2 Hz, 1H), 3.47 (s, 3H), 3.36 (like-t, 2H), 3.11 (s, 1H), 3.08 (t, J = 9.7 Hz, 2H), 2.46-2.70 (m, 4H), 2.11-2.22 (m, 1H), 1.92-2.09 (m, 4H), 1.71-1.78 (m, 1H), 1.62-1.71 (m, 1H), 1.48-1.60 (m, 3H), 1.43 (s, 9H); ^{13}C NMR ($CDCl_3$, 151 MHz): δ ppm 162.11, 159.18, 156.67, 154.49, 138.87, 128.11, 126.82, 114.26, 113.28, 103.49, 94.45, 79.69, 68.42,

56.27, 54.56, 53.43, 49.02, 39.13, 35.77, 29.86, 28.54, 27.66, 25.79, 25.21, 17.27, 17.19. ESI-MS: $[M+1]^+$, 528.84.

2-(tert-butoxycarbonyl)-1-(2-(8-methoxy-2-oxo-2H-chromen-3-yl)ethyl)decahydro-1H-pyrido[3,2,1-ij][1,6]naphthyridine 7(3a1H)-oxide **(5k, $C_{28}H_{38}N_2O_6$)**

White foam; 90% yield form 5e; ^{1}H NMR ($CDCl_3$, 600 MHz): δ ppm 7.49 (s, 1H), 7.15 (t, J = 7.9 Hz, 1H), 6.98-7.02 (m, 2H), 5.02 (br s, 1H), 4.12 (t, J = 12.0 Hz, 1H), 3.93 (s, 3H), 3.59 (dd, J = 5.2 Hz, 12.0 Hz, 1H), 3.28 (like-t, 2H), 3.15 (s, 1H), 3.08 (t, J = 11.4 Hz, 2H), 2.46-2.72 (m, 4H), 2.12-2.22 (m, 1H), 1.92-2.06 (m, 4H), 1.62-1.76 (m, 2H), 1.48-1.57 (m, 3H), 1.43 (s, 9H); ^{13}C NMR ($CDCl_3$, 151 MHz): δ ppm 161.43, 156.74, 147.04, 142.86, 139.10, 130.15, 124.17, 120.38, 118.91, 112.57, 79.81, 69.13, 68.90, 68.34, 56.31, 54.54, 48.97, 39.10, 35.74, 29.75, 28.62 (3C), 27.76, 25.79, 25.26, 17.34, 17.24. ESI-MS: $[M+1]^+$,498.83.

Synthesis of derivatives 5l~5q

The products **5** (1.5 mmol) were dissolved in MeOH/AcOEt (15mL/15mL) and Hydrogen chloride gas was bubbled through the solution for 1 hours at room temperature. The solvent was removed under reduced pressure, and the solid residue was recrystallized form methanol/ethanol in appropriate proportion to give the desired products.

3-(2-(decahydro-1H,4H-pyrido[3,2,1-ij][1,6]naphthyridin-1-yl)ethyl)-2H-chromen-2-one **(5l, $C_{22}H_{28}N_2O_2$)**

White solid; 60% yield from 5a; ^{1}H NMR (DMSO-d6, 600 MHz): δ ppm 11.07 (m, 1H), 9.75 (m, 1H), 9.40 (br d, J = 9.6 Hz, 1H), 7.98 (s, 1H), 7.68 (d, J = 7.7 Hz, 1H),

7.53-7.58 (m, 1H), 7.38 (d, J = 8.3 Hz, 1H), 7.31-7.36 (m, 1H), 4.18 (m, 1H), 3.82 (br q, J = 12.1 Hz, 1H), 3.57 (d, J = 10.4 Hz, 1H), 3.22 (m, 2H), 3.08-3.14 (br d, J = 12.1 Hz, 1H), 2.85-2.96 (m, 2H), 2.58-2.72 (m, 2H), 2.48 (overlap 1H), 2.23 (br d, J = 12.0 Hz, 1H), 2.02-2.11 (m, 1H), 1.94-2.00 (m, 1H), 1.81-1.94 (m, 2H), 1.58-1.80 (m, 6H); ^{13}C NMR (DMSO-d6, 151 MHz): δ ppm 160.8, 152.6, 139.9, 131.04, 128.0, 127.2, 124.5, 119.2, 115.9, 60.5, 54.5, 54.4, 51.0, 41.9, 35.4, 30.6, 27.8, 25.5, 24.1, 23.0, 18.0, 17.8. ESI-MS: $[M+1]^+$, 352.85.

3-(2-(decahydro-1H,4H-pyrido[3,2,1-ij][1,6]naphthyridin-1-yl)ethyl)-6-methyl-2H-chromen-2-one **(5m, $C_{23}H_{30}N_2O_2$)**

White solid; 60% yield from 5b; ^{1}H NMR (DMSO-d6, 600 MHz): δ ppm 10.96 (m, 1H), 9.78 (m, 1H), 9.43 (br d, J = 9.4 Hz, 1H), 7.90 (s, 1H), 7.45 (d, J = 1.5 Hz, 1H), 7.35 (dd, J = 1.5 Hz, 8.5 Hz, 1H), 7.26 (d, J = 8.5 Hz, 1H), 4.08 (m, 1H), 3.78 (br q, J = 12.2 Hz, 1H), 3.59 (d, J = 11.1 Hz, 1H), 3.21 (m, 2H), 3.07-3.14 (br d, J = 12.2 Hz, 1H), 2.85-2.96 (m, 2H), 2.57-2.70 (m, 2H), 2.50 (overlap 1H), 2.33 (s, 3H), 2.24 (br d, J = 12.4 Hz, 1H), 2.01-2.09 (m, 1H), 1.92-1.99 (m, 1H), 1.79-1.92 (m, 2H), 1.57-1.78 (m, 6H); ^{13}C NMR (DMSO-d6, 151 MHz): δ ppm 161.47, 151.28, 140.34, 134.10, 132.35, 128.15, 127.63, 119.44, 116.15, 60.98, 54.94, 54.84, 51.56, 42.37, 35.85, 31.02, 28.36, 26.07, 24.54, 23.49, 20.78, 18.47, 18.30. ESI-MS: $[M+1]^+$, 366.67.

3-(2-(decahydro-1H,4H-pyrido[3,2,1-ij][1,6]naphthyridin-1-yl)ethyl)-7-hydroxy-2H-chromen-2-one **(5n, $C_{22}H_{28}N_2O_3$)**

White solid; 58% yield from 5d; ^{1}H NMR (DMSO-d6，600 MHz): δ ppm 10.94 (br s, 1H), 10.58 (br s, 1H), 9.68 (br s, 1H), 9.40 (br s, 1H), 7.84 (s, 1H), 7.48 (d, J = 8.6 Hz,

1H), 6.77 (dd, J = 2.2 Hz, 8.6 Hz, 1H), 6.71 (d, J = 2.2 Hz, 1H), 4.05 (br s, 1H), 3.70-3.85 (m, 1H), 3.57 (br s, 1H), 3.21 (m, 2H), 3.04-3.14 (m, 1H), 2.80 (m, 2H), 2.52-2.60 (m, 2H), 2.48 (overlap 1H), 2.15-2.25 (m, 1H), 1.97-2.06 (m, 1H), 1.90-1.97 (m, 1H), 1.78-1.90 (m, 2H), 1.50-1.78 (m, 6H); ^{13}C NMR (DMSO-d6, 151 MHz): δ ppm 161.78, 161.01, 154.96, 140.92, 137.10, 130.13, 129.70, 113.62, 112.09, 102.30, 60.96, 60.23, 55.41, 55.40, 54.97, 54.88, 54.85, 31.15, 29.49, 28.62, 27.02, 25.72. ESI-MS: [M+1]$^+$, 368.67.

3-(2-(decahydro-1H,4H-pyrido[3,2,1-ij][1,6]naphthyridin-1-yl)ethyl)-7-methoxy-2H-chromen-2-one **(5o, $C_{23}H_{30}N_2O_3$)**

White solid; 45% yield from 5h; ^{1}H NMR (DMSO-d6，600 MHz): δ ppm 10.99 (br s, 1H), 9.87 (m, 1H), 9.43 (br s, 1H), 7.90 (s, 1H), 7.57 (d, J = 8.6 Hz, 1H), 6.94-6.97 (m, 1H), 6.90-6.93 (m, 1H), 4.07 (br s, 1H), 3.71-3.84 (m, 4H), 3.59 (br s, 1H), 3.20 (br s, 2H), 3.07-3.13 (m, 1H), 2.84-2.96 (m, 2H), 2.56-2.67 (m, 2H), 2.50 (overlap, 1H), 2.24 (br s, 1H), 1.99-2.07 (m, 1H), 1.92-1.98 (m, 1H), 1.79-1.92 (m, 2H), 1.52-1.80 (m, 6H); ^{13}C NMR (DMSO-d6, 151 MHz): δ ppm 162.23, 161.64, 154.89, 140.64, 129.50, 123.86, 113.20, 112.83, 100.84, 60.97, 56.36, 54.96, 54.85, 51.52, 42.37, 35.92, 31.12, 28.51, 25.81, 24.54, 18.49, 18.31. ESI-MS: [M+1]$^+$, 352.60.

6-chloro-3-(2-(decahydro-1H,4H-pyrido[3,2,1-ij][1,6]naphthyridin-1-yl)ethyl)-2H-chromen-2-one **(5p, $C_{22}H_{27}ClN_2O_2$)**

White solid; 61% yield from 5f; ^{1}H NMR (DMSO-d6，600 MHz): δ ppm 10.80-11.12 (m, 1H), 9.25-9.92 (m, 2H), 7.94-7.98 (m, 1H), 7.80 (s, 1H), 7.58-7.63 (m, 1H), 7.42-7.46 (m, 1H), 4.10 (br s, 1H), 3.79 (br q, J = 12.2 Hz, 1H), 3.55-3.62 (m, 1H),

3.23 (m, 2H), 3.10-3.17 (br d, J = 12.2 Hz, 1H), 2.86-2.97 (m, 2H), 2.59-2.73 (m, 2H), 2,45 (overlap, 1H), 2.17-2.29 (m, 1H), 2.03-2.12 (m, 1H), 1.93-2.20 (m, 1H), 1.80-1.93 (m, 2H), 1.58-1.80 (m, 6H); ^{13}C NMR (DMSO-d6, 151 MHz): δ ppm 160.90, 151.78, 139.21, 131.10, 128.99, 128.63, 127.58, 121.13, 118.41, 60.97, 55.39, 54.95, 54.85, 51.45, 35.93, 31.09, 28.21, 26.12, 24.54, 23.50, 18.48, 18.31. ESI-MS: $[M+1]^+$, 386.61.

6-bromo-3-(2-(decahydro-1H,4H-pyrido[3,2,1-ij][1,6]naphthyridin-1-yl)ethyl)-2H-chromen-2-one **(5q, $C_{22}H_{27}BrN_2O_2$)**

White solid; 50% yield from 5g; ^{1}H NMR (DMSO-d6，600 MHz): δ ppm 11.00-11.13 (m, 1H), 9.85-9.97 (m, 1H), 9.40 (br d, J = 9.7 Hz, 1H), 7.96 (s, 1H), 7.93 (d, J = 2.4 Hz, 1H), 7.71 (dd, J =2.4 Hz, 8.8 Hz, 1H), 7.37 (d, J = 8.8 Hz, 1H), 4.12 (m, 1H), 3.82 (br q, J = 12.2 Hz, 1H), 3.60 (d, J = 10.4 Hz, 1H), 3.25 (m, 2H), 3.09-3.15 (br d, J = 12.2 Hz, 1H), 2.86-2.97 (m, 2H), 2.60-2.74 (m, 2H), 2.53 (overlap, 1H), 2.27 (br d, J = 11.7 Hz, 1H), 2.03-2.11 (m, 1H), 1.82-1.99 (m, 3H), 1.59-1.80 (m, 6H); ^{13}C NMR (DMSO-d6, 151 MHz): δ ppm 160.84, 152.19, 139.14,133.84, 130.58, 128.93, 121.62, 118.68, 116.46, 60.98, 54.93, 54.84, 51.47, 42.37, 35.89, 31.00, 28.18, 26.21, 24.54, 23.49, 18.46, 18.30. ESI-MS: $[M+1]^+$, 432.55.

Supplementary figures

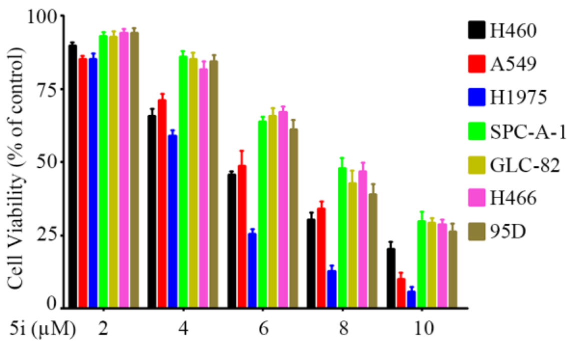

Figure S1. Cell viability assays of 5i on lung cancer cell lines.

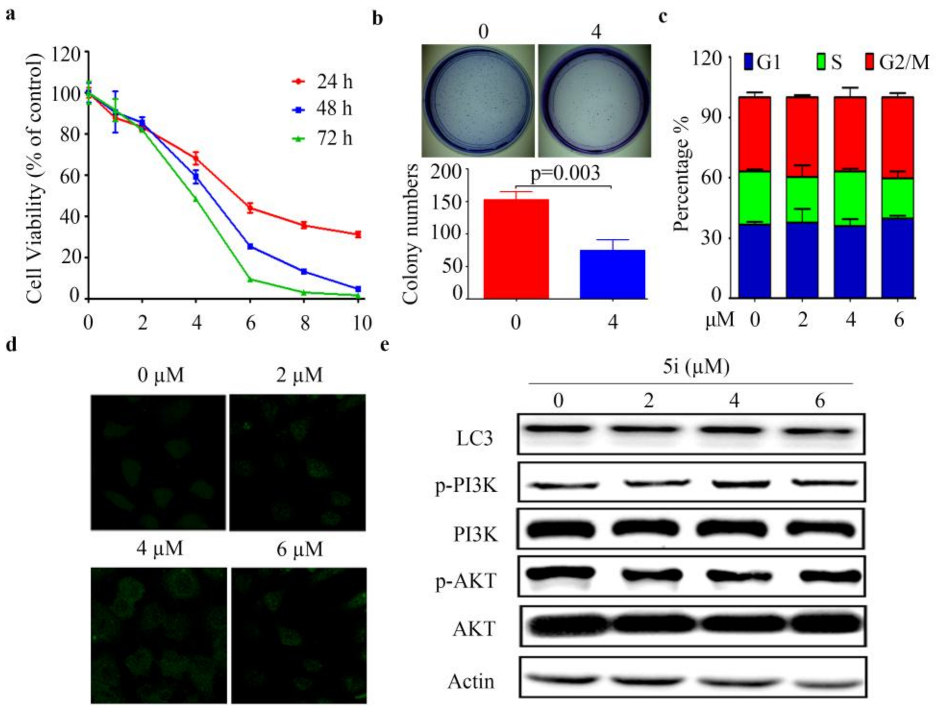

Figure S2. Compound 5i inhibited cell proliferation but could not induce G1 cell cycle arrest, autophagy and attenuate *PI3K/AKT* signaling pathway in H1975 lung cancer cells. (a) H1975 cells were treated with different concentrations of **5i** for indicated time points and assessed by trypan blue exclusion analysis. (b) Soft-agar colony formation assay for H1975 cells treated with or without **5i**. (c) **5i** could not induce G1 accumulation in H1975 cells. (d) H1975 cells were treated with **5i** for 24 hours and assessed by immunofluorescence analysis using an anti-LC3 antibody. (e)

5i could not activate LC3 and attenuate *PI3K/AKT* signaling pathway in H1975 cells.

Data are represented as mean $\pm$ SD and p value was calculated with t-test.
